# A Case of Subacute Stent Thrombosis

**DOI:** 10.7759/cureus.37725

**Published:** 2023-04-17

**Authors:** Ivan A Mijares-Rojas, Enrique F Martinez, George L Leonor Lopez, Eduardo De Marchena, Carlos E Alfonso

**Affiliations:** 1 Internal Medicine, University of Miami Miller School of Medicine, Jackson Memorial Hospital, Miami, USA; 2 Internal Medicine, John H. Stroger, Jr. Hospital of Cook County, Chicago, USA; 3 Cardiology, University of Miami Miller School of Medicine, Jackson Memorial Hospital, Miami, USA; 4 Cardiology, University of Miami Hospital, Miami, USA

**Keywords:** drug eluting stent, primary percutaneous coronary intervention (pci), intravascular ultrasound (ivus), coronary artery angiography, stent thrombosis

## Abstract

A 67-year-old male presenting with an anterior ST-segment elevation myocardial infarction (STEMI) underwent stent placement in the left anterior descending coronary. The patient was discharged on an appropriate medical regimen containing dual antiplatelet therapy (DAPT). Four days later, the patient presented with repeat acute coronary syndrome symptomatology. Electrocardiogram demonstrated ongoing STEMI in the previously treated artery distribution. Emergency angiography revealed restenosis and total thrombotic occlusion. Post-intervention stenosis was 0% after aspiration thrombectomy and balloon angioplasty. Stent thrombosis is a high-mortality and therapeutically challenging condition requiring prepared clinicians who recognize predisposing risk factors and initiate early management.

## Introduction

Stent thrombosis (ST) is the most feared complication after percutaneous coronary intervention (PCI). Recent analyses show a mortality rate of up to 45% and a recurrence rate of 15-20% at five years [1]. The introduction of first-generation drug-eluting stents (DES) drastically reduced the rates of in-stent restenosis compared to bare-metal stents but brought safety concerns regarding ST. Second-generation DES (G2-DES) has proved to be efficacious in preventing restenosis and lowering ST rates [2]. Nevertheless, ST still occurs, and ongoing research is elucidating the contributing factors to its development and novel strategies for its prevention. We present a case of subacute stent thrombosis in which early recognition led to appropriate management.

## Case presentation

**Figure 1:**
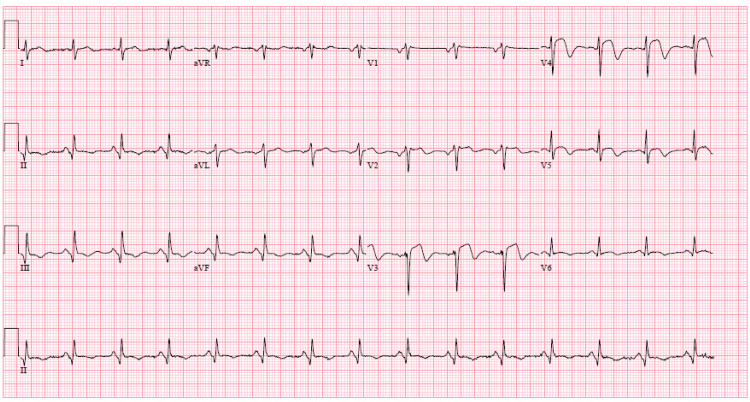
STEMI ECG. ECG consistent with ST-segment elevation in the anterior precordial leads V2-V4. STEMI: ST-segment elevation myocardial infarction; ECG: electrocardiogram.

A 67-year-old male was brought to our facility by emergency services as a ST-segment elevation myocardial infarction (STEMI) alert. He complained of typical chest pain and endorsed hypertension and current smoking as his only relevant past medical history. Arrival electrocardiogram (ECG) revealed an ongoing anterior infarct with ST-segment elevation in precordial leads V2-V4 (Figure 1).

**Figure 2:**
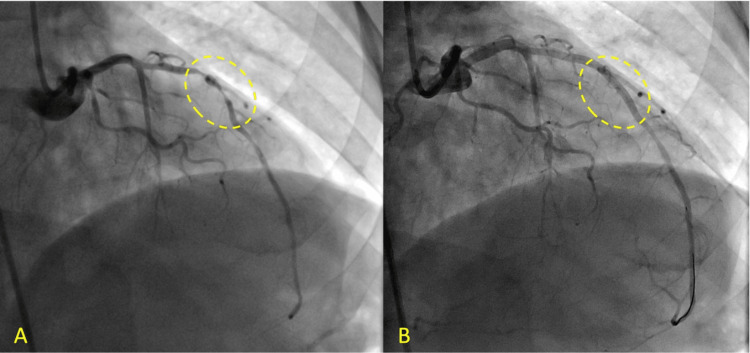
Angiography on STEMI presentation. (A) 90% stenosis within the middle portion of the LAD artery (dashed oval) with TIMI flow 2. (B) Status post stent deployment (dashed oval) with 0% stenosis and TIMI flow 3. STEMI: ST-segment elevation myocardial infarction; LAD: left anterior descending; TIMI: thrombolysis in myocardial infarction.

The patient had already been loaded with 325 mg aspirin by emergency services and received nitroglycerin for pain relief. Immediate heparin infusion with 4000 units bolus infusion, 180 mg ticagrelor, and 80 mg atorvastatin were started on his way to the catheterization lab. PCI demonstrated a 90% stenosis within the middle portion of the left anterior descending (LAD) artery with thrombolysis in myocardial infarction (TIMI) flow 2. The lesion was managed with balloon pre-dilation with a 2.5 x 20 mm balloon and a 2.75 x 26 mm Resolute-Onyx® (Medtronic, Minneapolis, MN, USA) stent deployment. TIMI flow 3 and 0% stenosis were achieved afterward (Figure 2).

Post-procedure ECG revealed moderately reduced left ventricle (LV) systolic function (calculated ejection fraction of 35-40%) with akinetic apex and anteroseptal wall. The patient remained stable and was discharged on a regimen of atorvastatin, carvedilol, losartan, and dual antiplatelet therapy (DAPT) of aspirin and ticagrelor.

**Figure 3:**
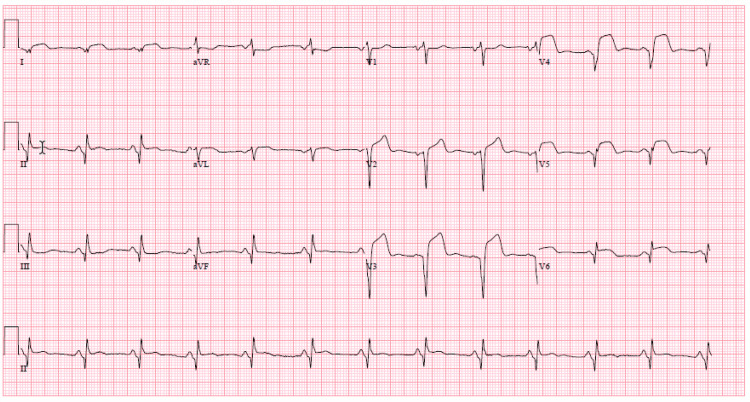
ECG on ST presentation. ECG consistent with ST-segment elevation to anterolateral leads I, aVL, V2-V6. ECG: electrocardiogram; ST: stent thrombosis.

Four days later, the patient presented with acute onset chest pain with identical characteristics and intensity as the last episode. He reported compliance with the discharge regimen. ECG revealed similar findings to the previous admission (Figure 3).

**Figure 4:**
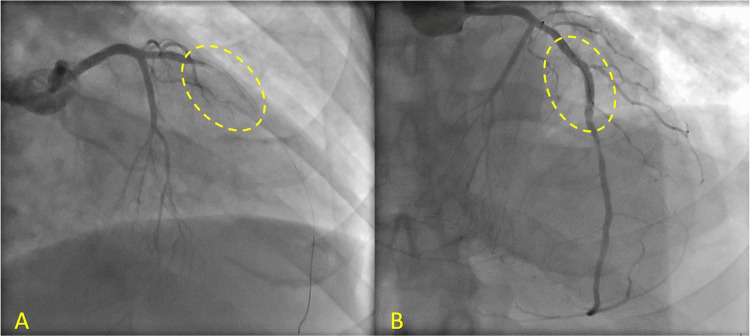
Angiography on ST presentation. (A) Total thrombotic occlusion of the pretreated segment (dashed oval) with TIMI flow 0. (B) Status post revascularization with aspiration thrombectomy and balloon angioplasty (dashed oval) with TIMI flow 3. ST: stent thrombosis.

The patient was immediately started on the acute coronary syndrome (ACS) protocol and transferred to the catheterization lab. Angiography demonstrated in-stent restenosis and total thrombotic occlusion of the previously treated segment with TIMI flow 0. Aspiration thrombectomy and balloon angioplasty were performed, achieving post-intervention stenosis of 0% and TIMI flow 3 (Figure 4).

**Figure 5:**
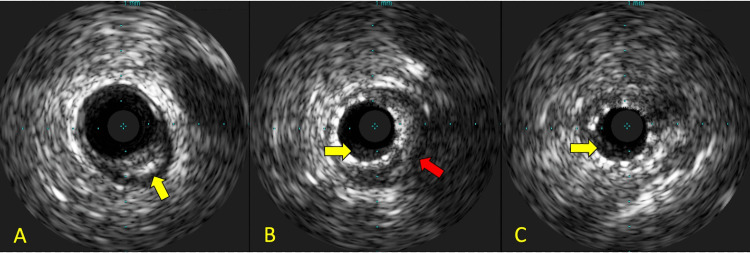
IVUS after achieving revascularization. (A) Segment immediately proximal to stent implantation demonstrates atheromatous plaque (yellow arrow). (B) The proximal stent segment demonstrates in-stent restenosis and residual thrombus within (yellow arrow) and behind the stent (red arrow). (C) Distal stent segment with stenosis and residual thrombus within the stent (yellow arrow). IVUS: intravascular ultrasound.

Post-PCI intravascular ultrasound (IVUS) showed mild residual thrombus (Figure 5), and the decision was made to start tirofiban infusion. The patient remained hemodynamically stable after the procedure, with a repeat ECG demonstrating similar cardiac function. On discharge, DAPT continuation for at least 12 months was indicated.

## Discussion

Since 2007, the Academic Research Consortium has categorized ST according to its timing after stent implantation as acute (first 24 hours), subacute (>24 hours to 30 days), late (>30 days to one year), or very late (>1 year). The condition's certainty level is differentiated as definite or probable based on angiographic or postmortem pathological confirmation for ischemic syndromes in the pretreated vessels [2,3]. G2-DES development aimed to address the upsurge in the incidence of ST events seen with first-generation DES. This goal was achieved through changes in stent composition, with an improved thinner strut in conjunction with novel biocompatible or biodegradable polymers [2]. Recent studies have demonstrated decreased incidence rates with G2-DES, particularly in the late and very late ST timeframe presentation [4,5]. Nonetheless, acute and subacute ST continue to be a burden, with an incidence as high as 4.9% among patients with STEMI undergoing PCI [6].

Analyses have described the multifactorial pathophysiology of ST, including patient-, device-, lesion-, and procedure-related predictors contributing to its development [1,2,7]. Although all predictors play a role, we now have a better idea of which factors are most critical in tilting the balance toward an early versus a late presentation. For example, late and very late ST presentations appear pathophysiologically related to chronic inflammatory states, with patients on hemodialysis, patients with diabetes, and patients with malignancy being the most commonly affected [1,7]. Hence, it is unsurprising that introducing novel biocompatible stent materials and upgraded immunomodulators in G2-DES lowered its occurrence [8]. Conversely, acute and subacute ST are mainly associated with patient- and procedure-related factors. Patients who present with ACS, develop cardiogenic shock, have a left ventricle ejection fraction <40%, have diabetes, or smoke show a higher risk [1,2,7]. Given the increased platelet activation and ongoing endothelialization, suboptimal DAPT or DAPT nonresponsiveness can be catastrophic and provoke early ST [7]. Stent underexpansion has been identified as the most crucial procedural factor in some cohorts [1,2,6,7]. Left main coronary artery or LAD artery lesions are also highly predisposed, particularly if severely calcified or with a TIMI flow <3 [1,6,7].

Although ST is a widely recognized complication, its prognosis and management remain understudied. Notably, ST patients have a fourfold higher incidence of in-hospital death and cardiovascular complications [9]. Some authors have attributed this to a higher thrombus burden, more complex baseline characteristics, and recurrent ST among these patients compared to patients with de novo ACS [1,2,4]. Current management guidelines are extrapolated from ACS protocols and emphasize the need for acute PCI for revascularization [10]. Given its reported mortality benefit, a set goal of TIMI flow 3 must be aggressively pursued, taking advantage of aspiration and distal protective devices if needed [11]. After revascularization, applying novel intravascular imaging devices (optical coherence tomography, IVUS) may help elucidate fixable pathologic underlying mechanisms [2,9]. When suspected, antiplatelet nonresponsiveness should be tested with measurement of platelet inhibition or detection of cytochrome P450 2C19 polymorphism [2,12].

## Conclusions

Even though the case presented demonstrated an excellent result with an aggressive approach, we must emphasize that prevention is the first line of therapy for ST. From the results obtained with the IVUS, we can hypothesize that stent underexpansion and the multiple risk factors in this particular patient (presentation, type, and location of the lesion) could have predisposed the occurrence of subacute ST. The future state-of-the-art PCI will include appropriate risk stratification, optimal antiplatelet regimen, accessibility to intravascular imaging, and proper post-procedural patient education. Clinicians may soon make use of it, improving the outcomes of patients with the highest risk of the spectrum. 
